# Pass/Fail Quality Assessment in Last Mile Smart Metering Networks Based on PRIME Interface

**DOI:** 10.3390/s21227444

**Published:** 2021-11-09

**Authors:** Piotr Kiedrowski, Beata Marciniak

**Affiliations:** Faculty of Telecommunications Computer Science and Electrical Engineering, Bydgoszcz University of Science and Technology, Al. Prof. S. Kaliskiego 7, 85-796 Bydgoszcz, Poland; Beata.Marciniak@pbs.edu.pl

**Keywords:** industrial IoT, PRIME, smart metering, wired sensor networks

## Abstract

The pass/fail form is one of the presentation methods of quality assessment results. The authors, as part of a research team, participated in the process of creating the PRIME interface analyzer. The PRIME interface is a standardized interface—considered as communication technology for smart metering wired networks, which are specific kinds of sensor networks. The frame error ratio (FER) assessment and its presentation in the pass/fail form was one of the problems that needed to be solves in the PRIME analyzer project. In this paper, the authors present their method of a unified FER assessment, which was implemented in the PRIME analyzer, as one of its many functionalities. The need for FER unification is the result of using different modulation types and an optional forward error correction mechanism in the PRIME interface. Having one unified FER and a threshold value makes it possible to present measurement results in the pass/fail form. For FER unification, the characteristics of FER vs. signal-to-noise ratio, for all modulations implemented in PRIME, were used in the proposed algorithm (and some are presented in this paper). In communication systems, the FER value is used to forecast the quality of a link or service, but using PLC technology, forecasting is highly uncertain due to the main noise. The presentation of the measurement results in the pass/fail form is important because it allows unskilled staff to make many laborious measurements in last mile smart metering networks.

## 1. Introduction

New measurement techniques solutions are the natural “consequences” of implementing new technologies in communication services. This is also true for communication systems that are designed for remote reading of electrical energy consumption. In most cases, smart metering (SM) systems use wireless [1,2] or power line communications (PLC) [3,4] technologies for last mile network realization. PLC is gaining popularity [5] among other technologies due to its standardization by ITU-T [6,7,8]. These standards define such PLC interfaces as: PoweRline intelligent metering evolution (PRIME), G3-PLC [9], and G.hnem [10]. These interfaces are based on orthogonal frequency division multiplexing (OFDM) as physical layers [11] and differential phase shift keying (DPSK) as carrier modulations. For the PRIME interface, they are: differential binary phase shift keying (DBPSK) modulation, differential quaternary phase shift keying (DQPSK) modulation, and differential 8-phase-shift keying (D8PSK) modulation. The PRIME interface, as with all interfaces used in SM, uses carrier frequencies within the CENELEC A band [9,12], i.e., from 42 to 89 kHz. The authors of this paper constructed a PLC PRIME analyzer to meet the expectations of smart metering maintenance personnel.

Analyzers used for measurements in communication networks have similar constructions. Nowadays, they consist of microcomputers with a touch screen and a specialized module. In case of PLC, the specialized module is a standard PLC modem. If PLC technology is used for SM systems, the same modem that is used in the traffic concentrator (TC) can be deployed. Depending on the used modem, dedicated software must be implemented based on the conformity with the host modem interface documentation. This software is a modem driver.

Further software components of the PLC PRIME analyzer are: implementation of ITU-T G.9904 protocols, a quality assessment module, a graphical user interface, a reports creator, a remote manager, a statistic data processor, and presenter. The presented methods were implemented on a Raspberry Pi microcomputer using Python 3 programming language. This paper focuses on a specific form of statistic data presentation—pass/fail. The pass/fail presentation form allows unskilled staff to make measurements in a low voltage (LV) power network. This method of presentation is useful in a fault localization. The most common fault source, which degrades the communication quality in last mile SM networks based on PLC, is the capacitive load.

## 2. Problem Definition

Typical interface analyzers consist of three functional parts: a protocols analyzer, a physical layer tester, and a quality monitor. The quality monitor uses statistics data for assessing the communication reliability in a particular layer. For the quality assessment in physical and data layers, these statistic data involve the frame error ratio (FER). The FER depends on the signal-to-noise ratio (SNR) [13]. There are different characteristics of FER vs. SNR for different kinds of modulations [14], length of frames, and whether a forward error correction (FEC) technique is deployed. According to ITU-T recommendation G.9904, the convolutional coding (CC) method [15] is used as a FEC realization for PRIME. The value of FER is defined as:

(1) $$\mathrm{FER}=\frac{f_{e}}{f_{Tx}},$$

where: $f_{e}$ is the number of received erroneous frames, $f_{Tx}$ is the number of sent frames.

In the monitoring mode, the value of $f_{Tx}$ is unknown, because frames are generated by SMs and TC, and not by the analyzer. In such a case, the value of FER is obtained by the following formula:

(2) $$\;\mathrm{FER}=\frac{f_{e}}{f_{e}+f_{ef}},$$

where $f_{ef}$ is the number of received error free frames.

The frame is classified as erroneous when the cyclic redundancy check (CRC) [16] mismatches. CRC-8 and CRC-32 are used in the PRIME interface, the CRC field may be placed at the end of the frame or at the end of the frame overhead. Considering that, the PRIME frame may have a length in the range of 18 to 2268 bytes (the upper range depends on the type of modulation). There are three types of modulation; for every kind of modulation, the FEC can be on or off. There are thousands of options, in regard to FER vs. SNR characteristics. Thus, many observation results are difficult to present or interpret; another main problem is the duration of the observation, which is required to obtain measurement results with the resolution, at least $10^{-2}$ for each type of FER. Of course, we can use the data obtained from the frames of rare lengths, but the problem is still not solved, especially as the network analyzer dedicated for technical staff should have so-called easily interpretable result presentation forms, e.g., pass/fail form. The pass/fail presentation method depends on the comparison of the resulting value with the threshold constant value. The software implementation of the result presentation in the form of past/fail is not difficult if only threshold constant values are known; the difficulty is posed by the unification of the FER vs. SNR characteristics to one characteristic, which may act as a reference characteristic. The solution to this problem and the problem of the proposed method evaluation is also discussed in this paper.

## 3. The Method for Unified FER Assessment

According to the PRIME specification [8], the value of SNR is delivered from the PLC modem by $PHY_{-}SNR.confirm$ primitive as result of sending the $PHY_{-}SNR$.get primitive to the modem. The semantics of this primitive are as follows: $PHY_{-}SNR.confirmSNR$.

The SNR parameter refers to the signal-to-noise ratio, defined as the ratio of the measured received signal level to noise level of the last received PHY protocol data unit (PPDU) [8]. It may take one of eight values. The mapping of the three-bit index to the actual SNR value is given below:

0: ≤0 dB
1: ≤3 dB and >0 dB
2: ≤6 dB≤ and >3 dB
⋯
7: >18 dB.

To create and present the communication performance for a particular kind of modulation, two matrices are declared: the matrix of received erroneous frame counters and the matrix of all received frame counters. The size of the matrix is 2250×8, because there are 2250 possible frame lengths and eight ranges of SNRs. According to (2), for the given kind of modulation and frame length, FER is:

(3) $$\mathrm{FER}=\frac{\sum_{i=1}^{8}e_{i,k}}{\sum_{i=1}^{8}a_{i,k}},\;a\in N^{+}$$

where $e_{k}$ are error counters (eight elements in the matrix of received erroneous frames), $a_{k}$ are all frame counters (eight elements in the matrix of all received frame counters), *k* is the index of frame length.

**Figure 1:**
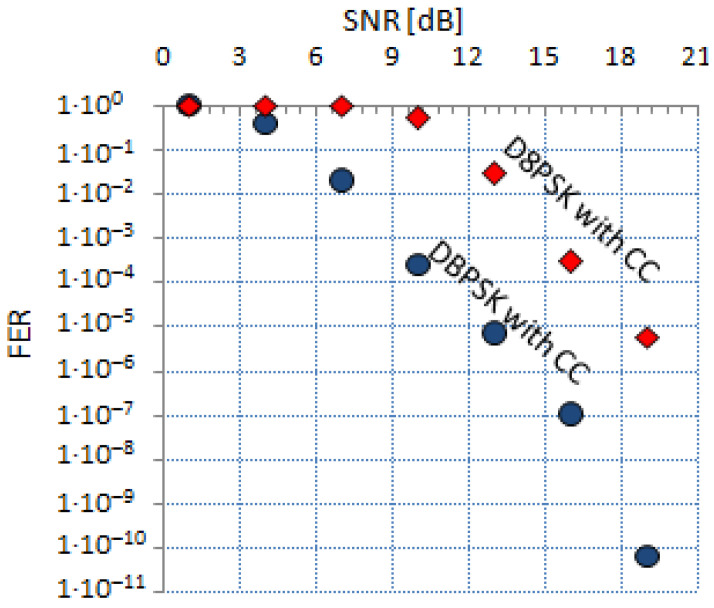
FER vs. SNR characteristics, for DBPSK with CC, and D8PSK with CC modulations, performed with the frame size of 18 bytes.

Such data organization also allows to create FER(SNR) characteristics, as done in [17], using simulation methods, or in [18], using a virtual lab methodology; we used the data obtained from real, long time measurements. FER(SNR) characteristics presented in the graph format are called performance curves, and for the same frame length, they differ, depending on the type of modulation. As an example, two characteristics of FER(SNR), for DBPSK with CC, and D8PSK with CC modulations, are presented in Figure 1.

**Figure 2:**
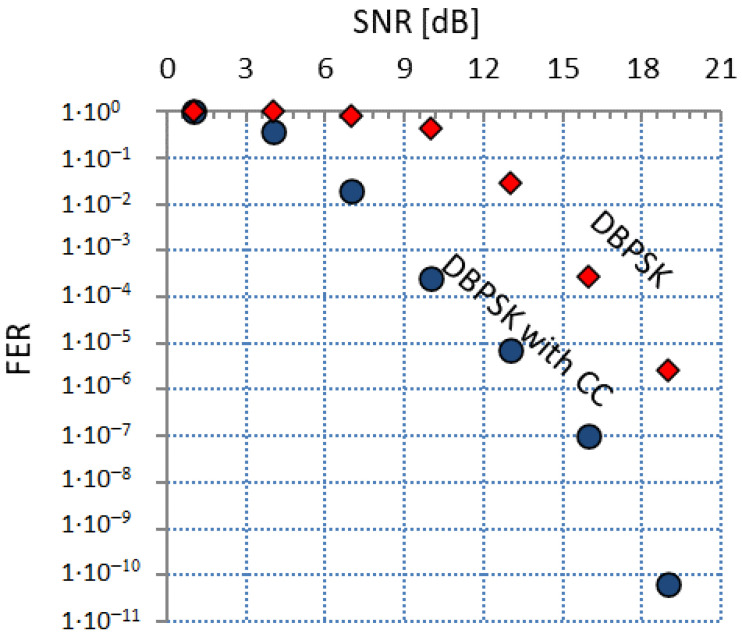
FER vs. SNR characteristics, for DBPSK with CC and DBPSK without CC modulations, performed with a frame size of 18 bytes.

The characteristics of FER(SNR) also allows to assess the robustness of the modulation, e.g., using the data presented in Figure 1, we can conclude that DBPSK with CC is more robust than D8PSK with CC. Due to the same value of SNR, the level of FER is smaller for DBPSK with CC. The effectiveness of the FEC, which is based on the CC method, is illustrated in Figure 2, where two characteristics of FER(SNR) for DBPSK and DBPSK with CC are presented.

Three characteristics for 18-byte frames are presented in Figure 1 and Figure 2. For two types of modulation (e.g., *A* and *B*) and for the same constant value of SNR, the equivalent of ${\mathrm{FER}}_{A}$ calculated over the number of erroneous and error free frames modulated with the use of *B* modulation may be expressed as follows:

(4) $${\mathrm{FER}}_{A}=\alpha_{AB}\left(\mathrm{SNR}\right)\cdot {\mathrm{FER}}_{B}\left(\mathrm{SNR}\right)$$

where $\alpha_{AB}\left(\mathrm{SNR}\right)$ is a FER conversion factor, to express FER as if frames were modulated with use of the *A* modulation, while in fact they were modulated with use of the B modulation.

The value of $\alpha_{AB}\left(\mathrm{SNR}\right)$ is the quotient of ${\mathrm{FER}}_{A}\left(\mathrm{SNR}\right)$ and ${\mathrm{FER}}_{B}\left(\mathrm{SNR}\right)$, i.e., $\alpha_{AB}=1/\alpha_{BA}$. The values of FER, which are used to determine the conversion factor, must come from long-term measurement results, e.g., these presented in Figure 1 or Figure 2. Below, in Table 1, we present the values of $\alpha_{DBPSKcc_{-}D8PSKcc}$ factors obtained from the data presented in Figure 1.

The values of conversion factors presented in Table 1 are not bigger than 1. It is because $\alpha_{DBPSKcc_{-}D8PSKcc}$ is the factor used to express the FER of the more robust modulation using the data obtained from the less robust modulation. If it were opposite, the conversion factors would not be less than 1. Thus, using (4), for conversion FER, from the more robust modulation to FER, of the less robust modulation, we must remember that FER cannot be bigger than 1. In practice, we always convert to the most robust modulation (that is DBPSK with CC) from data obtained from all used by the PRIME modulation. Using the data from more than one type of modulation, we cannot use (4) or average them. In such a case, we use Formula (5), which includes the numbers of received frames per modulation type. Using the same variables, which were used in (3), we can express the value of one unified FER for the different types of modulations, and the particular length of frames, as follows:

(5) $${\mathrm{FER}}_{A}\left(k\right)=\frac{\sum_{i=1}^{8}\left(e_{Ai,k}+\alpha_{A•}e_{•i,k}\right)}{\sum_{i=0}^{8}\left(a_{Ai,k}+a_{•i,k}\right)}$$

where • denotes all types of modulations, except the *A* modulation, other designations, such as in (3).

The index A of FER means that this FER is equal to the FER value of the *A* modulation calculated from the data taken from six pairs of counters. To have one, the global FER value, which is independent from SNR, nominators, and denominators, were summed before dividing.

**Figure 3:**
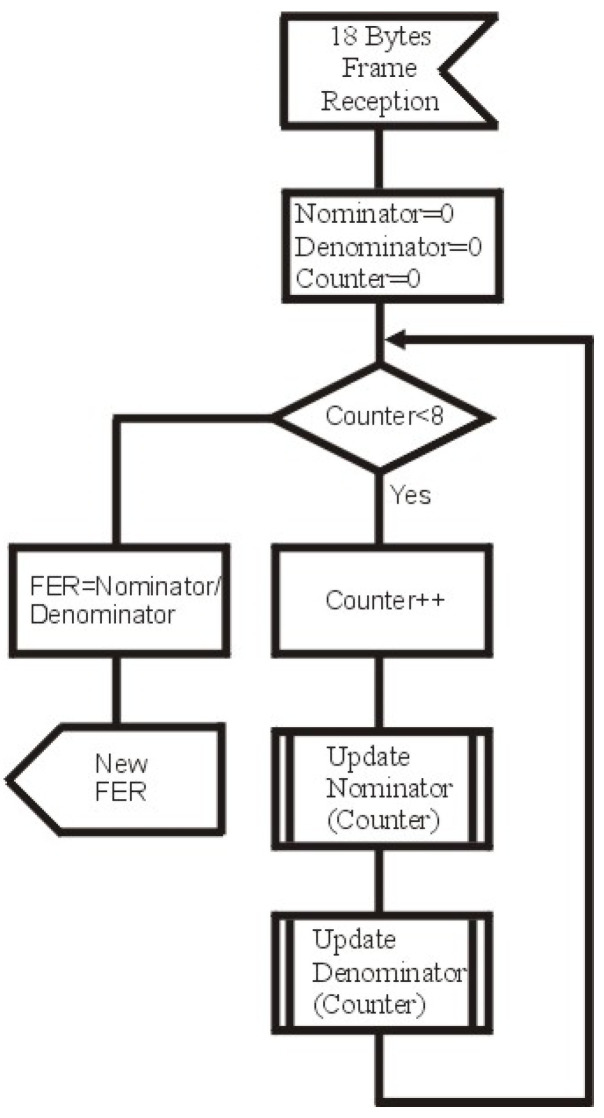
The algorithm for FER calculation.

The last problem is the influence of the frame length on FER, which is that, for the constant value of SNR, the FER increases as the frame length increases. We could use the same method that we used to become independent from the type of modulation, but there is a statistical problem. The FER is the probability of error occurrence determined from the sample, and when we receive very few frames of the specified lengths, even during a long-term observation, we can only consider this sample as insignificant. To solve this problem, we decided to only use two types of frames for the unified FER assessment: the “beacon” frame and the “promotion need MAC” frame. The “beacon” frame is transmitted very often—the frequency of the “beacon” frame generation is one “beacon” frame per $2^{n}$ other frames, where *n* may be: 0, 1, 2, 3, 4, or 5. The length of the “beacon” frame is fixed and equals 18 bytes, the last 4 bytes are CRC-32 field. The “promotion need MAC” frame length is also 18 bytes, but it ends with CRC-8. Based on the two types of frames with the same length, and the fact that there are two types of CRC, it could be possible to assess FER over CRC-32 and FER over CRC-8, but it is not necessary due to the fact that they give the same values of FER. The algorithm for the FER calculation for the pass/fail presentation is presented in Figure 3, in sequence diagram language form.

The presented algorithm based on Formulas (4) and (5) has two procedures: UpdateNominator and UpdateDenominator; the argument of both procedures is the Counter value. Nominator and denominator variables are global variables, similar to conversion factors. Sample codes of the bodies of these procedures are presented below, respectively.


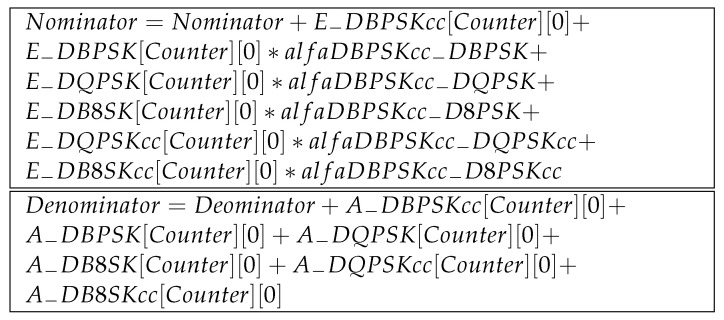


Counters matrices have two indexes, the first index is the variable counter, whilst the second is 0, because 18-byte frames are the shortest ones. The $E_{-}DBPSKcc$ and $A_{-}DBPSKcc$ matrices are also used in the FER self-assessment evaluation method, which is described in the next section.

## 4. The Self-Evaluation of Measurement Reliability

**Figure 4:**
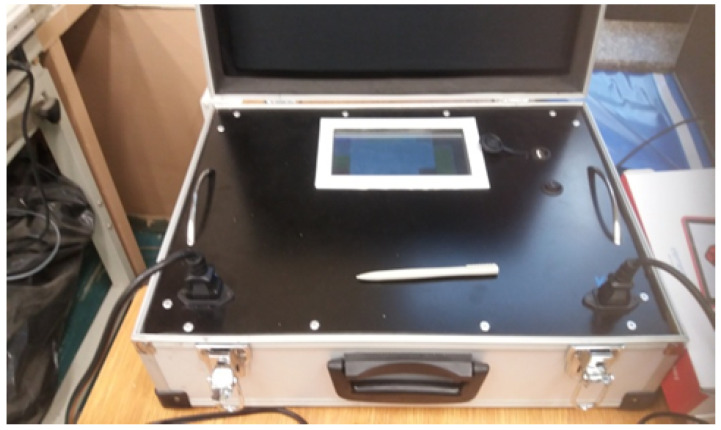
Photograph of the PRIME analyzer α-prototype.

The proposed method of the unified FER assessment was implemented on a Raspberry Pi microcomputer, which, together with PLC PRIME modem, separate circuits, three-phase line interface, and a piece of software created in Python 3, constitute an embedded system named PRIME analyzer. A photograph of the prototype of this analyzer is presented in Figure 4.

One of the functionalities of the PRIME analyzer presented in Figure 4 was to enable unskilled staff to make serial measurement sessions with the purpose of finding fault sources. The localization of the fault source is carried out with the use of the unified FER value compared to the configured $FER_{-}threshold$ value. If FER is greater than $FER_{-}threshold$, then the test is failed; in other case, it is passed. The $FER_{-}threshold$ value is not configurable from the level of GUI, it is only configurable from the operation system by a system administrator. For the evaluation of the implemented method of the FER assessment and to ensure a sufficiently long observation time, the current, measured FER(SNR) values are compared with eight reference values of $FER_{-}DBPSKcc$. The eight values in the $FER_{-}DBPSKcc$ array are centrally distributed after they are updated. The data obtained during the measurements are used to update the already existing values—they are cumulated. This way, the FER(SNR) values are more accurate, because we have more statistical material. There is uncertainty about the use of FER vs. SNR charts in both measuring and quality forecasting. The nature of the noise is the source of the uncertainty. The noise level is determined over time, so it is only an average level of it. FER vs. SNR charts for the same kind of modulation can differ if the noise level changes rapidly. This phenomenon is especially noticeable when the power line supplies gas-discharge lamps [19]. After the process of data validation, it is distributed to PRIME analyzers. The processes of data acquisition and distribution are realized during the report creation process, when the analyzer is connected to the internet. The data that are presented in Figure 1 and Figure 2 come from the central base, which was updated on 29 November 2020. The $FER_{-}DBPSKcc$ array is used in the self-evaluation process during the measurement; additionally, the array $E_{-}DBPSKcc$ and $A_{-}DBPSKcc$ matrices are used. The process of self-evaluation is to ensure the reliability of the measurement and is realized using the following formula:

(6) $$\delta =\frac{1}{8}\sum_{i=0}^{7}\frac{|\mathrm{FER}\left[i\right]-\frac{e[i][0]}{a[i][0]}|}{\mathrm{FER}[i]},$$

where: *i* is an SNR index in matrices and array, FER represents $FER_{-}DBPSKcc$ array, *e* represents $E_{-}DBPSKcc$ matrix, and *a* represents $A_{-}DBPSKcc$ matrix.

**Figure 5:**
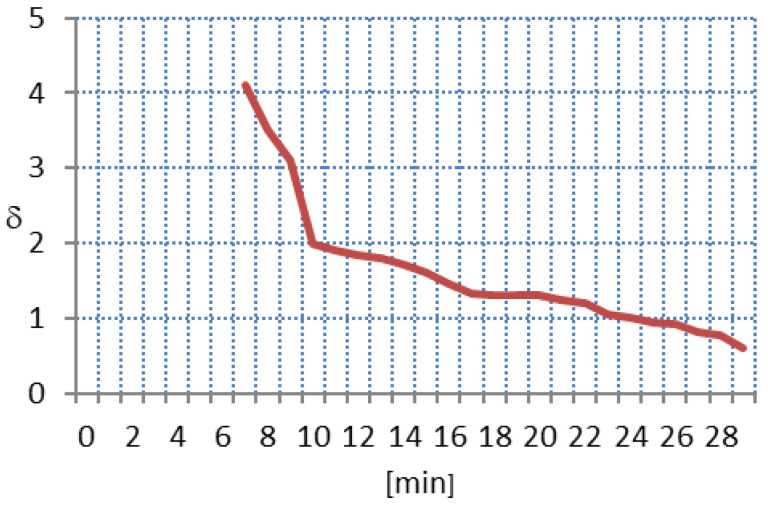
Exemplary process of δ value determination.

The above Formula (6) is nothing but a mean value of the absolute error. The evaluation process starts if all eight values $A_{-}DBPSKcc\left[•\right]\left[0\right]$ are different from zero. Typically, the duration of a pending state is a few minutes. After this time, the calculation of δ value can be done. If δ < 1, the pass/fail FER assessment may start. As an example, in Figure 5, the process of the δ value determination is shown over time.

It can be observed from Figure 5 that the duration of the pending state is 7 min. This is typical, because every 15 min [20] (or sometimes 30 min) [21], the reading process of all SMs in the last mile network starts, which causes the communication traffic increase.

The duration of the pending state will be shorter if the SMs are read more frequently, which is likely to happen in the near future. It is shown in [22] that frequent readings allow describing household consumption profile features with greater accuracy. The need for more frequent readings can also be caused by the development of distributed generation and energy storage systems. The advantages of the frequent PV household profile readings are shown in [23].

## 5. Discussion

The proposed unified FER assessment method allows for the realization that most expert systems are easier, because there is only one value for consideration. The deployment of this method in the pass/fail testing mode allows unskilled staff to make measurements in an LV network (obviously without neglecting any of the safety requirements). Several minutes of testing of the last mile network is becoming more popular, because of the so-called “unintentional attacks”. The most common source of these attacks is the incompatible or damaged load connection to the mains. The second type source of an “unintentional attack” could be the high-capacity load.

The FER values that depend on the value of the capacitance of the capacitor connected to the power line are presented in Table 2.

**Figure 6:**
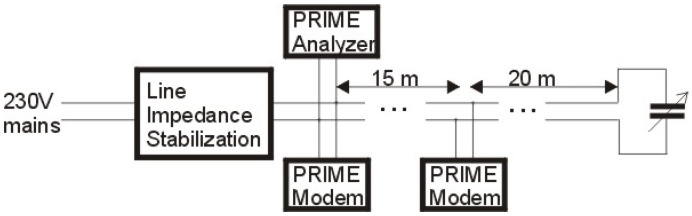
Schema of the circuit used for FER vs. capacitance characteristic obtaining.

The data presented in Table 2 are the results of laboratory tests. The schema of the electrical circuit used for the tests is shown in Figure 6.

We used a line impedance stabilization network produced by Rohde and Schwarz to separate our circuit from the mains noises and its impedance. PRIME modems were used for the traffic generation and PRIME analyzer was used to measure the FER. In our experiment, we used a 10 mm${}^{2}$ copper solid conductor cable as a power line. The distance of 15 m between the modems was too small to affect the transmission quality, while the distance of 20 m between the second modem and the capacitor is the typical average distance between the SM and the power consumer’s load. The distance between the measuring point and the capacity influences the measurement result—the longer the distance, the smaller the FER. This feature allows locating a household with a capacitive receiver (the source of an “unintentional attack”). The presence of the capacity causes the signal transmitted by the modem to be attenuated, which reduces the SNR value. The reduction in the SNR value may also be caused by noise, which also increases the FER value. To determine whether the source of transmission errors is capacitance or noise, the presented PRIME analyzer analysis a shape of the PRIME frame preamble. The PRIME frame preamble is the linear chirp signal. The preamble duration is 2048 μs, the start frequency is 41,992 Hz, and the final frequency 88,867 Hz, so the level of the preamble signal weakens over time, which is when the capacitance is the source of error rather than noise. Preamble analysis is performed by specialized personnel only if the pass/fail test result is a fail.

The proposed method allows localizing the source of these “attacks”; moreover, further analysis makes it possible to determine if the source has a capacitive or disturbing character. The analysis of the SNR values, together with the signal levels of receiving frames, are useful during the determination of the fault source character. The proposed self-evaluation method ensures the measurement reliability and makes the pass/fail verdict not hasty. It should also be mentioned that there are some areas of implementation where the proposed method is practically inefficient. These areas are small, last mile networks. Small networks are the networks with a few SMs located in close proximity to TC. In such networks, it is impossible to receive frames with all eight possible SNRs, which cause the self-evaluation process to stay in the pending state. This disadvantage is not particularly bothersome, because in small networks, faults are rare. Sporadic dysfunctions of small last mile networks (which do not allow reading of SMs) have no particular effect on the quality of the statistical material, which is used by the distributed system operators to electrical power consumption forecasting [24].

## Figures and Tables

**Table 1:** $\alpha_{DBPSKcc_{-}D8PSKcc}$ conversion factors for 18-byte frames.

SNR [dB]	≤0	(0, 3]	(3, 6]	(6, 9]	(9, 12]	(12, 15]	(15, 18]	>18
$\alpha_{DBPSKcc_{-}D8PSKcc}$	1	1	0.39	0.02	$4.7\times 10^{-4}$	$3.5\times 10^{-4}$	$2.5\times 10^{-4}$	$1\times 10^{-5}$

**Table 2:** FER vs. the capacitance located near the measuring point.

Capacitance	0.5 μF	0.9 μF	1.4 μF	1.9 μF	2.6 μF	2.8 μF	3.3 μF	3.8 μF	4.2 μF	4.8 μF
**FER**	0	0	0.05	0.13	0.37	0.56	0.81	0.94	1	1

## Data Availability

Data available directly from the authors.

## Abbreviations

The following abbreviations are used in this manuscript:

FER	frame error ratio: the number of frame errors divided by the total number of transferred frames
SM	smart metering
PLC	power line communication
PRIME	PoweRline Intelligent Metering Evolution
DPSK	differential phase shift keying
DQPSK	differential quaternary phase shift keying
D8PSK	differential 8 phase shift keying
CENELEC	European Committee for Electrotechnical Standardization
ITU-T	Telecommunication Standardization Sector
SNR	signal-to-noise ratio
FEC	forward error correction
CRC	cyclic redundancy check
PPDU	PHY protocol data unit
SDL	sequence diagram language
